# *Pseudomonas fluorescens* Filamentous Hemagglutinin, an Iron-Regulated Protein, Is an Important Virulence Factor that Modulates Bacterial Pathogenicity

**DOI:** 10.3389/fmicb.2016.01320

**Published:** 2016-08-23

**Authors:** Yuan-Yuan Sun, Heng Chi, Li Sun

**Affiliations:** ^1^Key Laboratory of Experimental Marine Biology, Institute of Oceanology – Chinese Academy of SciencesQingdao, China; ^2^Laboratory for Marine Biology and Biotechnology, Qingdao National Laboratory for Marine Science and TechnologyQingdao, China; ^3^University of Chinese Academy of SciencesBeijing, China

**Keywords:** *Pseudomonas fluorescens*, filamentous hemagglutinin, motility, adhesion, virulence, vaccine

## Abstract

*Pseudomonas fluorescens* is a common bacterial pathogen to a wide range of aquaculture animals including various species of fish. In this study, we employed proteomic analysis and identified filamentous hemagglutinin (FHA) as an iron-responsive protein secreted by TSS, a pathogenic *P. fluorescens* isolate. *In vitro* study showed that compared to the wild type, the *fha* mutant TSS*fha* (i) exhibited a largely similar vegetative growth profile but significantly retarded in the ability of biofilm growth and producing extracellular matrix, (ii) displayed no apparent flagella and motility, (iii) was defective in the attachment to host cells and unable to form self-aggregation, (iv) displayed markedly reduced capacity of hemagglutination and surviving in host serum. *In vivo* infection analysis revealed that TSS*fha* was significantly attenuated in the ability of dissemination in fish tissues and inducing host mortality, and that antibody blocking of the natural FHA produced by the wild type TSS impaired the infectivity of the pathogen. Furthermore, when introduced into turbot as a subunit vaccine, recombinant FHA elicited a significant protection against lethal TSS challenge. Taken together, these results indicate for the first time that *P. fluorescens* FHA is a key virulence factor essential to multiple biological processes associated with pathogenicity.

## Introduction

For many bacterial pathogens, attachment to host tissues by various adhesins is the critical step in the process of infection (Pizarro-Cerda and Cossart, 2006). Adhesins are a large family of proteins that includes fimbriae (Romantschuk, 1992; Ojanen-Reuhs et al., 1997; Shakhnovich et al., 2007), type IV pili (Roine et al., 1998; Poole et al., 2007), flagella (Roy et al., 2009), trimeric autotransporter adhesins (Mil-Homens and Fialho, 2012; Mikula et al., 2013), and filamentous hemagglutinin (FHA).

Filamentous hemagglutinin is an important adhesin present both in a secreted and surface-associated form in *Bordetella* (Weiss and Hewlett, 1986; Locht et al., 1993; Jacob-Dubuisson et al., 2000). One FHA that has been extensively characterized is from *Bordetella pertussis*. It is secreted in a Sec-dependent manner by the two-partner secretion (TPS) system, which includes an outer membrane-associated accessory protein interacting with FHA N-proximal secretion domain. Functionally, it has proapoptotic activity and promotes bacterial aggregation (Locht et al., 1993, 2001; Romero et al., 2014). In *Xanthomonas axonopodis* pv. *citri*, a FHA-like protein is required for tissue colonization, surface attachment, cell-to-cell aggregation and biofilm formation (Gottig et al., 2009). Another FHA-like protein in *Acinetobacter baumannii* plays a role in virulence in a mouse lethal model of infection, promoting biofilm formation and mediating the adhesion of *A. baumannii* to epithelial cells (Astaneh et al., 2014). Apart from its role as an adhesin, FHA of *B. pertussis* and *Bordetella bronchiseptica* also possesses immunomodulatory properties which may contribute to subversion of host innate and adaptive immunity (Abramson et al., 2001; Braat et al., 2007; Julio et al., 2009; Henderson et al., 2012; Romero et al., 2014).

*Pseudomonas fluorescens* is a Gram-negative bacterium existing widely in soil, water, plant, and animals. In aquaculture, it is a common pathogen for shrimp and a wide range of fish species (Swain et al., 2007; Wang et al., 2009). In addition, *P. fluorescens* can also infect humans and is known to cause outbreaks of bacteremia (Gershman et al., 2008). Unlike environmental *P. fluorescens* from water and soil, pathogenic *P. fluorescens* from fish have been studied on a very limited base. In this study, with an aim to gain new insight into the infection mechanism of *P. fluorescens*, we investigated the function of *P. fluorescens* FHA in an infection model of turbot (*Scophthalmus maximus*).

## Materials and Methods

### Ethics Statement

Experiments involving live animals were conducted in accordance with the “Regulations for the Administration of Affairs Concerning Experimental Animals” promulgated by the State Science and Technology Commission of Shandong Province. The study was approved by the ethics committee of Institute of Oceanology – Chinese Academy of Sciences.

### Bacterial Strains and Growth Conditions

*Pseudomonas fluorescens* TSS is a pathogenic fish isolate that has been reported previously (Wang et al., 2009). *Escherichia coli* BL21(DE3) and DH5α were purchased from TransGen Biotech (Beijing, China). *E. coli* S17-1 λpir was purchased from Biomedal. All strains were grown in Luria-Bertani broth (LB) at 37°C (for *E. coli*) or 28°C (for TSS). Where indicated, 2,2′-dipyridyl (Sigma, St. Louis, MO, USA), tetracycline, and chloramphenicol were added at the concentrations of 600 μM, 20 and 50 μg/ml, respectively.

### Two-Dimensional Gel Electrophoresis (2-DE), MALDI-TOF/TOF-MS and Protein Identification

Two-dimensional gel electrophoresis, MALDI-TOF/TOF-MS, and protein identification were performed as reported previously (Liu et al., 2015).

### Sequence Analysis

The sequence of *P. fluorescens fha* has been reported previously (GenBank accession no. WP_014719704.1). The amino acid sequence was analyzed using the BLAST program at the National Center for Biotechnology Information (NCBI) and the Expert Protein Analysis System. Domain search was performed with the conserved domain search program of NCBI. Subcellular localization prediction was performed with the PSORTb v.3.0 server.

### Construction of TSS*fha* and TSSΔ*fha*

In order to create TSS*fha*, we first created the plasmid p705T, which was used for gene mutagenesis, as follows: the tetracycline-resistance gene of p704T (Hu et al., 2009) was cut out by SmaI/ScaI double digestion and inserted into pGP704 (Miller and Mekalanos, 1988) at the ScaI site. To construct the *Pf_Fha_*-defective strain TSS*fha*, the internal fragment of *Pf_Fha_* (positions 241–408) was amplified by PCR with the primer pairs F (5′-AGATCTGTGGTGTTGAACAACGCCT-3′, underlined se-quence, BglII site) and R (5′-AGATCTATCGGCCGCCTGGCCGAA-3′, underlined sequence, BglII site). The PCR product was inserted into the suicide plasmid p705T at the compatible BglII site, resulting in p705Fha. S17-1 λpir was transformed with p705Fha, and the transformant was conjugated with *P. fluorescens* TSS as described previously (Sun et al., 2009). The transconjugant was selected on LB agar plates supplemented with tetracycline and chloramphenicol, and one of the resistant clones was named TSS*fha*. Mutation of *Pf_Fha_* in TSS*fha* was confirmed by PCR analysis. In addition, single-copy plasmid insertion in TSS*fha* was further confirmed by the quantitative real-time PCR (qRT-PCR) method described previously (Zhang et al., 2014).

To construct TSSΔ*fha*, in-frame deletion of a 582 bp segment (positions 40–621) of *Pf_Fha_* was performed by overlap extension PCR as follows: the first overlap PCR was performed with the primers F2 (5′-CCCGGGAACTGGCCTACAAAGACGT-3′, underlined sequence, SmaI site) and R2 (5′-CGACCTTCCTGGGGTGAAAGGTGGA-3′), the second overlap PCR was performed with the primers F3 (5′-CACCCCAGGAAGGTCGCCTCAGTGCTCG-3′) and R3 (5′-CCCGGGGGTGATGCTGCGTTGTTCG-3′, underlined sequence, SmaI site), and the fusion PCR was performed with the primer pair F2/R3. The PCR products were inserted into the suicide plasmid p7TS (Wang et al., 2009) at the SmaI site, resulting in p7TSFha. p7TSFha was introduced into S17-1 λpir (Biomedal, Spain) by transformation. The transformant S17-1 λpir/p7TSFha was conjugated with TSS. The transconjugants were selected first on LB plates supplemented with tetracycline and chloramphenicol and then on LB plates supplemented with 12% sucrose and chloramphenicol. The colonies that appeared on the plates were analyzed by PCR, and the PCR products were subjected to sequence analysis to confirm deletion of *Pf_Fha_*.

### Adhesion to FG Cells

FG-9307 cells, a cell line established from Japanese flounder gill cells (Tong et al., 1997), were cultured at 22°C in 96-well cell culture plates (∼10^5^ cell/well) with L-15 medium (GIBCO, Invitrogen, Carlsbad, CA, USA) as described previously (Tong et al., 1997). TSS and TSS*fha* were cultured in LB medium to an OD_600_ of 0.8 and were resuspended in L-15 medium to 1 × 10^7^ CFU/ml. One hundred microliter of bacterial suspension was added to FG cells cultured as above. The plates were incubated at 28°C for 1, 2, and 4 h, followed by washing three times with PBS. FG cells were then lysed with 1% Triton X-100, and 50 μl lysate was plated in triplicate on LB agar plates. The plates were incubated at 28°C for 48 h, and the colonies that appeared on the plates were enumerated. The genetic identity of the colonies was verified by PCR and sequence analysis of selected PCR products. The experiment was performed three times.

### Autoaggregation and Production of Extracellular Matrix

For autoaggregation analysis, TSS and TSS*fha* were cultured at 28°C in test tubes containing LB broth in a shaking incubator to an OD_600_ of 0.8. The tubes were removed from the shaker, and 100 microliters of cell suspension were added into 96-well microplates and incubated overnight. Meanwhile, the static cultures in the test tubes were monitored for sedimentation for 48 h. Bacterial autoaggregation in microplates and glass tubes were then examined. For microscopy, TSS and TSS*fha* were cultured as above, 100 microliters of cell suspension were added into a 12-well plate with embedded coverslips for 8 h at 28°C. Autoaggregation of cells on coverslips were photographed with a scanning electron microscope (S-3400N, Hitachi, Japan; Wang and Sun, 2015). For extracellular matrix analysis, TSS and TSS*fha* were cultured as above, 100 microliters of cell suspension were added into a 12-well culture plate with embedded coverslips for 20 h at 28°C. The cells were then observed with a scanning electron microscope. All experiments were performed three times.

### Motility Assay and Flagella Formation

To measure motility, TSS, TSS*fha*, and TSSΔ*fha* were cultured in LB medium to an OD_600_ of 1.0, and 5 μl cell suspension were spotted onto the center of fresh swimming plates containing LB medium plus 0.3% (w/v) agar. The plates were then incubated at 28°C. Two days later, the motility of the bacteria was assessed by examining the diameter of the motility halo on the soft agar. To measure flagella formation, the bacteria were cultured in LB agar plates at 28°C for 20 h and examined with a transmission electron microscope (JEM-1200, Jeol, Japan) as reported previously (Sun and Sun, 2016). The assays were performed three times.

### Biofilm Formation Assay

Quantitative biofilm formation on polystyrene surfaces (96-well microtiter plates) was investigated as reported previously (Wang et al., 2013a). Briefly, 10^7^ CFU of TSS, TSS*fha*, and TSSΔ*fha* were placed into a sterile 96-well flat-bottomed tissue culture plate and incubated at 28°C for 12 h. After incubation, the plates were washed to remove unbound cells, and the bound cells were stained with crystal violet. Quantification of the bound cells was performed by measuring the dissolved crystal violet at OD_570_ after the addition of 100 μl 30% acetic acid. To quantify biofilm formation in glass tubes, the bacteria were cultured as above and the cultures in tubes were incubated under static conditions for 48 h at 28°C. Then the culture mediums were removed and the glass tubes were washed three times with PBS. Subsequently, 5 ml of 1% crystal violet dye was added to glass tube for 15 min at room temperature. Following, the tubes were washed three times with phosphate buffered saline (PBS). After addition of 5 ml of 30% acetic acid for 15 min at room temperature to solubilize the crystal violet, the glass tubes were allowed to dry and then photographed. The experiment was performed three times.

### Hemagglutination Assay

TSS and TSS*fha* cultured as above were resuspended in PBS to 2 × 10^9^, 2 × 10^8^, 2 × 10^7^, and 2 × 10^6^ CFU/ml respectively. Turbot red blood cells were collected, washed three times in PBS, and resuspended to a final concentration of 2% (v/v). Fifty microliters of bacterial suspension or PBS (control) was mixed with 50 μl of turbot erythrocytes in a 96-well microtiter V-bottom plate. The plate was incubated for 1 h at room temperature. Hemagglutination was detected by visual inspection as reported previously (Astaneh et al., 2014). A small pellet of erythrocytes at the bottom of the well after incubation was considered negative as against positive reactions exhibiting an even sheet of erythrocytes across the wells. The experiment was performed three times.

### Serum Survival Assay

Serum survival analysis was performed as reported previously (Wang et al., 2013b).

### Tissue Infection and Mortality Assay

Clinically healthy turbot (average13.6 g) were purchased from a local fish farm. The fish were maintained as reported previously (Sun and Sun, 2015). For tissue dissemination and colonization analysis, TSS, TSS*fha*, and TSSΔ*fha* were cultured in LB medium to an OD_600_ of 0.8. The cells were washed with PBS and resuspended in PBS to 10^8^ CFU/ml. Turbot were divided randomly into four groups (*N* = 15) and infected by intramuscular (i.m.) injection with 50 μl of TSS, TSS*fha*, TSSΔ*fha*, or PBS. Kidney and spleen were taken from the fish at 12, 24, and 48 h post-infection (5 fish/time point). The tissues were homogenized in PBS with an OSE-20 electrictissue homogenizer (Tiangen, Beijing, China). The homogenates were diluted serially in PBS and plated in triplicate on LB agar plates. After incubation at 28°C for 48 h, the colonies that appeared on the plates were counted. The genetic identity of the colonies was verified as above. For mortality assay, turbot were infected with TSS, TSS*fha*, and PBS as above, and the fish were monitored for mortality over a period of 20 days. All experiments were performed three times.

### Purification of Recombinant Proteins and Antibody Preparation

The plasmids pEtFha, which expresses recombinant Pf_Fha_ (rFha), was constructed as follows. *Pf_Fha_* containing the hemag-glutination activity domain (positions 1–684) was amplified by PCR with the primer pairs F (5′-GATATCATGCCGACTACTCCACACAG-3′, underlined sequence, EcoRV site) and R (5′-GATATCGAAGTCGACCTGATTGCGG-3′, underlined sequence, EcoRV site). The PCR product was ligated with the T-A cloning vector pEASY-T1 Simple (TransGen Biotech, Beijing, China), and the recombinant plasmid was digested with EcoRV to retrieve the *Pf_Fha_*-containing fragment, which was inserted into pET259 (Hu et al., 2010) at the SwaI site, resulting pEtFha. To purify rFha and the control protein rTrx, *E. coli* BL21(DE3; TransGen Biotech, Beijing, China) was transformed with pEtFha and pET32a (Novagen, San Diego, CA, USA), the latter plasmid expresses Trx; the transformants were cultured in LB medium at 37°C to mid-logarithmic phase, and isopropyl-α-D-thiogalactopyranoside was added to the culture to a final concentration of 1 mM. After growing at 16°C for an additional 16 h, the cells were harvested by centrifugation, and His-tagged rFha and rTrx were purified using Ni-NTA Agarose (Qiagen, Valencia, CA, USA) as recommended by the manufacturer. The purified proteins were reconstituted, removed of endotoxin, and concentrated as described previously (Wang and Sun, 2015). Mouse antibody against rFha was prepared as reported previously (Sun and Sun, 2015). Control antibody from pre-immune mouse was also prepared. The antibody was purified using rProtein G Beads (Solarbio, Beijing, China). The specificity of the rFha antibody was determined by Western immunoblot as reported previously (Wang and Sun, 2015).

### Immunofluorescence Microscopy

To detect Pf_Fha_ on TSS, the bacteria were cultured in LB medium supplemented with 2,2′-dipyridyl as indicated above to OD_600_ 0.9; the cells were collected by centrifugation, washed with PBS, and resuspended in PBS to 1 × 10^8^ CFU/ml. One hundred microliters of bacterial suspension (∼1 × 10^7^ CFU) were seeded on a glass slide pre-treated with 0.001% polylysine (Sigma, St. Louis, MO, USA). For fluorescence microscopy, the samples were blocked with 1% bovine serum albumin for 2 h at 30°C and incubated with mouse anti-rFha antibody or control antibody from pre-immune mouse (1/500 dilution) for 1 h at 30°C. The cells were washed three times with PBST (0.05% Tween 20 in PBS) and incubated with goat anti-mouse IgG (1/1000 dilution; Bioss, Beijing, China) coupled to fluorescein isothiocyanate (FITC) for 1 h at 30°C. The cells were washed with PBST and stained with 4′,6-diamidino-2-phenylindole (DAPI) according to the instructions of the manufacturer (Bioss, Beijing, China). The cells were then analyzed with a Zeiss fluorescence microscope (Carl Zeiss Imager A2, Jena, Germany).

To examine interaction between rFha and FG cells, the cells were seeded on 0.001% polylysine-treated glass coverslips in 12-well cell culture plates; the cells were incubated with rFha or rTrx (100 μg/ml) at 22°C for 2 h and treated with paraformaldehyde (Sigma, St. Louis, MO, USA) at 22°C for 30 min. The cells were then treated with 1% bovine serum albumin at 4°C for overnight. The cells were incubated with mouse-anti His antibody (1/1000 dilution; Bioss, Beijing, China) for 1 h at 37°C. Subsequently, cells were washed three times with PBST and incubated with FITC-coupled goat-anti mouse IgG (1/1000 dilution; Bioss, Beijing, China) for 1 h at 37°C. After washing three times with PBST, the cells were stained with DAPI and subjected to microscopy as described above.

### Effect of Antibody Blocking on Bacterial Infection

Turbot (as described above) were divided randomly into three groups (*N* = 15). TSS was cultured in LB medium to an OD_600_ of 0.8. The cells were washed and resuspended in PBS to 10^8^ CFU/ml. One milliliter bacterial cells were mixed with 5 μl rFha antibody, control antibody, or PBS (control) and incubated at 28°C for 1 h. After incubation, 50 μl of the mixture was inoculated into turbot via i.m. injection. Kidney and spleen were taken from the fish (five at each time point) at 12, 24, and 48 h post-bacterial infection, and bacterial recovery from the tissues was determined as above.

### Immunization and Enzyme-Linked Immunosorbent Assay (ELISA)

rFha was resuspended in PBS to a concentration of 200 μg/ml and mixed at an equal volume with aluminum hydroxide as described previously (Jiao et al., 2010). As a control, PBS was mixed similarly with aluminum hydroxide without protein. Turbot (as described above) were divided randomly into two groups (*N* = 55) and injected intraperitoneally with 50 μl of the protein mixture or the PBS control. At 1 month post-immunization, the fish were challenged with TSS at the dose of 5 × 10^6^ CFU/fish. The fish were monitored for mortality for a period of 20 days. Dying fish were randomly selected for the examination of bacterial recovery from liver, kidney, and spleen. Relative percent of survival (RPS) was calculated according to the following formula: RPS = {1 – (% mortality in immunized fish/% mortality in control fish)}× 100. The immunization experiment was performed two times.

### Statistical Analysis

With the exception of the immunization trial which was performed twice, all other experiments were performed three times, and statistical analyses were carried out with the SPSS 17.0 package (SPSS, Inc., Chicago, IL, USA). Except for the survival analysis in the immunization experiment and the *in vivo* infection experiment, for which logrank test was used, analysis of variance (ANOVA) was used for all other analyses. In all cases, the significance level was defined as *P* < 0.05.

## Results

### Identification and Characterization of Pf_Fha_

**FIGURE 1:**
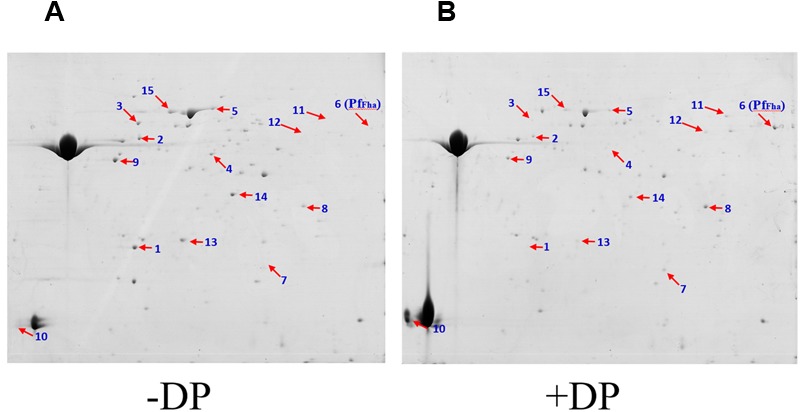
**Representative 2-DE maps of the extracellular protein profiles of *Pseudomonas fluorescens* TSS cultured under different conditions.** Extracellular proteins of *P. fluorescens* TSS cultured in the presence **(B)** and absence of 2,2′-dipyridyl (DP) **(A)** were subjected to 2-DE analysis. Numbers indicate protein spots with differential expression. The spot of Pf_Fha_ (number 6) is indicated.

In an effort to identify secreted proteins of TSS regulated by iron, TSS was cultured in the presence and absence of the iron chelator 2,2′-dipyridyl, and the extracellular proteins were examined by two-DE analysis. A total of 15 differentially expressed proteins were identified, of which six were significantly upregulated (ratio ≥ 2, *P* ≤ 0.05) in the presence of 2,2′-dipyridyl (**Figure 1**; Supplementary Table S1). Pf_Fha_ was one of the upregulated proteins (**Figure 1**). Sequence analysis showed that Pf_Fha_ contains an N-terminal signal peptide (residues 1–32) and a hemagglutination activity domain (residues 50–170; Supplementary Figure S1).

### *In vitro* Effect of *Pf_Fha_* Mutation

#### Cellular Motility, Flagella Formation, and Autoaggregation

**FIGURE 2:**
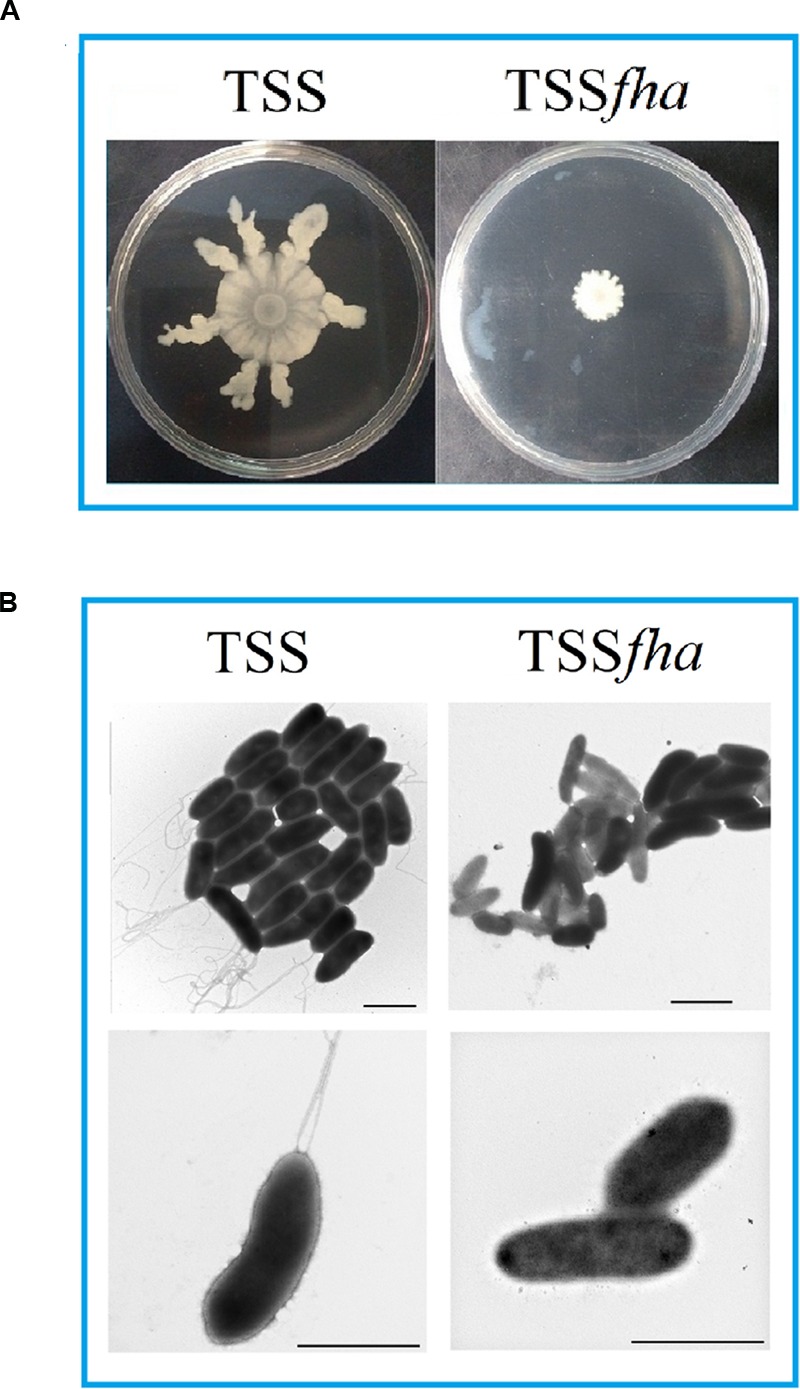
**Motility and flagella formation of *Pseudomonas fluorescens* TSS and TSS*fha*. (A)** TSS (left) and TSS*fha* (right) were cultured in LB medium to an OD_600_ of 1.0, and 5 μl cell suspensions were spotted onto the center of swimming plates containing LB medium plus 0.3% (w/v) agar. The plates were incubated at 28°C for 2 days. **(B)** TSS (left) and TSS*fha* (right) were cultured in LB agar plates and examined with a transmission electron microscope. Scale bar, 1 μm. The results represent one of three independent experiments.

**FIGURE 3:**
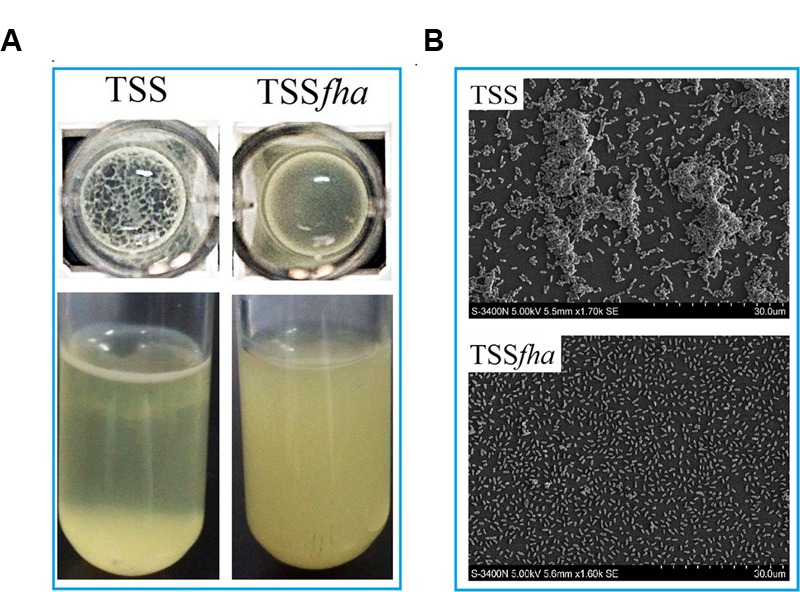
**Autoaggregation of *Pseudomonas fluorescens* TSS and TSS*fha*. (A)** TSS (left) and TSS*fha* (right) were cultured in LB medium at 28°C to an OD_600_ of 0.8; 100 microliters of cell suspensions were added into 96-well microplates and incubated overnight (Top), or the cell cultures were left standing for 2 days at room temperature without shaking (Bottom). **(B)** Autoaggregation observed by scanning electron microscope (SEM). TSS and TSS*fha* were cultured in LB medium at 28°C to an OD_600_ of 0.8; cell suspensions were added onto coverslips and observed by SEM after incubating for 8 h at room temperature. The pictures represent one of three independent experiments.

To examine the function of Pf_Fha_, a TSS mutant defective in *Pf_Fha_* was created based on insertion mutagenesis and named TSS*fha*. In addition, another *Pf_Fha_*-defective mutant, TSSΔ*fha*, was also created by markerless in-frame deletion. Compared to TSS, the motility of TSS*fha* in soft LB agar plate was almost abolished (**Figure 2A**). Similar observation was made with TSSΔ*fha* (Supplementary Figure S2A). Electron microscopy showed that while polar flagella were observed with the cells of TSS, no apparent flagella were observed with the cells of TSS*fha* (**Figure 2B**). Similar observation was made with TSSΔ*fha* (Supplementary Figure S2B). Cultured TSS cells were able to aggregate when left standing in the culture tube or being placed into a microplate, whereas, TSS*fha* failed to do so (**Figure 3A**). Consistently, scanning electron microscopy detected apparent aggregation of TSS cells but not TSS*fha* cells (**Figure 3B**).

#### Biofilm and Extracellular Matrix Production

**FIGURE 4:**
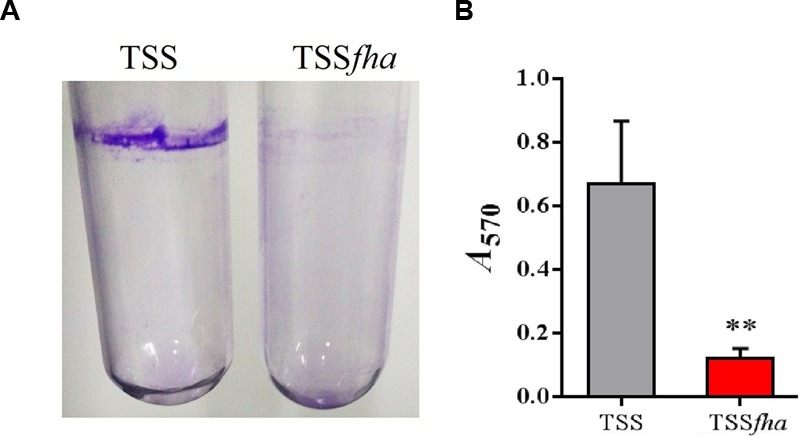
**Biofilm formation of *Pseudomonas fluorescens* TSS and TSS*fha*. (A)** Image of crystal violet (CV)-stained biofilms formed inside glass tubes. Cells were grown in LB medium overnight at 28°C and then stood for 2 days at room temperature before staining with CV. Image shown is from one representative experiment of three independent experiments. **(B)** Quantitation of biofilm. Stained biofilms were dissolved in 30% acetic acid, and the optical density at OD_570_ was recorded. Data are the means of three independent assays and presented as mean ± SEM. ^∗∗^*P* < 0.01.

**FIGURE 5:**
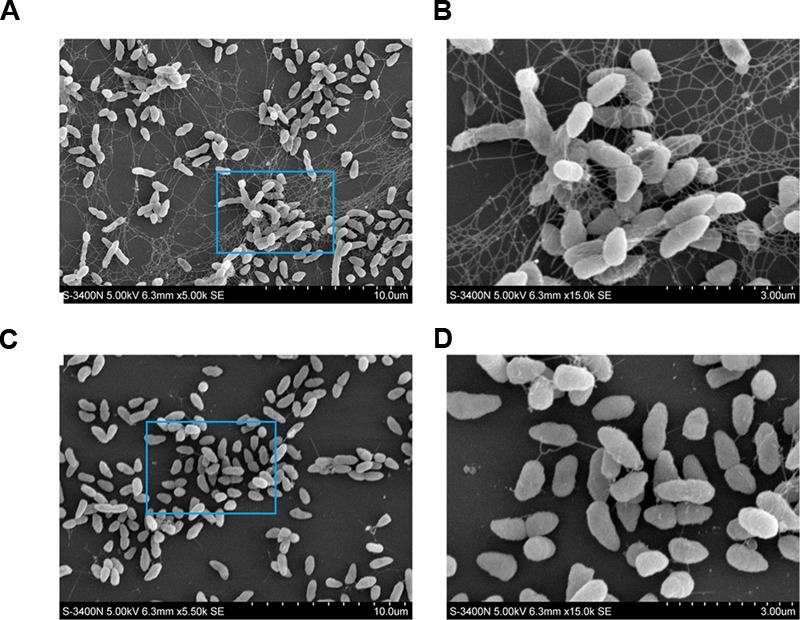
**Production of extracellular matrix structure by *Pseudomonas fluorescens* TSS and TSS*fha*.** TSS **(A,B)** and TSS*fha* **(C,D)** were cultured in LB medium at 28°C to an OD_600_ of 0.8; cell suspensions were added onto coverslips and observed by a scanning electron microscope after incubation for 20 h at room temperature. The results represent one of three independent experiments.

Vegetative growth analysis showed that when cultured in LB medium, TSS*fha* was comparable to TSS in growth profile, while when cultured in LB medium supplemented with 2,2′-dipyridyl, TSS*fha* grew slightly slower than TSS at the logarithmic phase and reached similar cell densities to TSS at the stationary phase (Supplementary Figure S3). The growth profile of TSSΔ*fha* was very similar to that of TSS*fha* (Supplementary Figure S3). Biofilm growth showed that TSS*fha* exhibited significantly reduced capacity to form biofilm (**Figure 4**). Similar observation was made with TSSΔ*fha* (Supplementary Figure S4). Scanning electron microscopy revealed that when cultured TSS cells were left on a coverslip for 20 h, a matrix of networks formed by fiber-like structures was observed abundantly among the cells, whereas very little such structure was formed by TSS*fha* (**Figure 5**).

#### Host Cell Adhesion, Hemagglutination, and Survival in Host Serum

**FIGURE 6:**
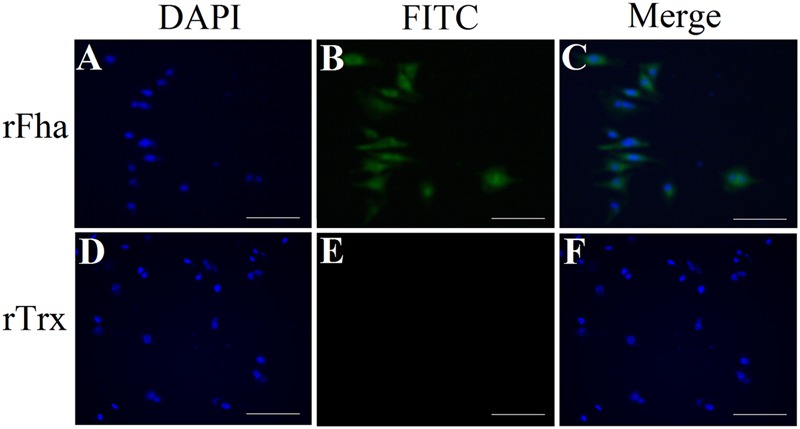
**Binding of rFha to FG-9307 cells.** FG cells were incubated with rFha **(A,B)** or the control protein rTrx **(D,E)**, and the cell-bound protein was detected with FITC-labeled antibody. The cells were stained with DAPI and observed with a fluorescence microscope. **(C)** A merge of **(A)** and **(B)**; **(F)** A merge of **(D)** and **(E)**. Magnifications: 20 × 10, scale bars: 50 μm.

Immunofluorescence microscopy showed that Pf_Fha_ was localized on the surface of the bacteria (Supplementary Figure S5). With this observation, we examined the potential involvement of Pf_Fha_ in host cell adhesion. The results showed that when incubated with flounder FG cells, the numbers of host cell-bound TSS increased with time; in contrast, the numbers of host cell-bound TSS*fha* barely changed and were 10.8-, 17.2-, and 60.8-fold lower than those of host cell-bound TSS at 1, 2, and 4 h of incubation (Supplementary Figure S6). These results suggested a requirement of Pf_Fha_ for host interaction. With this result, we further investigated whether Pf_Fha_ could bind directly to host cells. For this purpose, FG cells were incubated with recombinant Pf_Fha_ (rFha), which was purified as a His-tagged protein (Supplementary Figure S7), and cell-bound rFha was detected with immunofluorescence microscopy. The results showed that rFha was detected on FG cells (**Figure 6**). In contrast, when FG cells were similarly incubated with rTrx, the control protein purified under the same condition as rFha, no cell-bound protein was detected (**Figure 6**).

**FIGURE 7:**
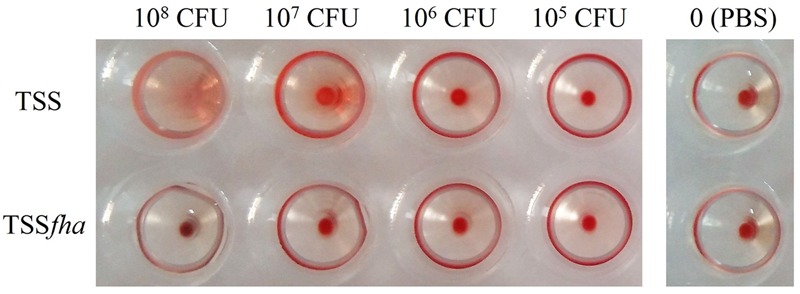
**Hemagglutination of *Pseudomonas fluorescens* TSS and TSS*fha*.** Turbot red blood cells were incubated with PBS (control) or with TSS and TSS*fha* in various concentrations for 1 h at room temperature, and hemagglutination was observed. The results represent one of three independent experiments.

Hemagglutination analysis indicated that incubation of TSS with turbot red blood cells induced agglutination of the blood cells in a manner that depended on the concentration of TSS; in contrast, no apparent hemagglutination was caused by TSS*fha* even at high concentrations (**Figure 7**). Serum survival analysis showed that following incubation with turbot serum for 1 h, TSS and TSS*fha* exhibited survival rates of 53.8 ± 10.3 and 30.1 ± 3.9%, respectively, the latter being significantly (*P* < 0.05) lower than that of the former. Similar to TSS*fha*, the survival rate of TSSΔ*fha* in turbot serum (28.3 ± 2.8%) was significantly (*P* < 0.05) lower than that of TSS.

### *In vivo* Effect of *Pf_Fha_* Mutation

**FIGURE 8:**
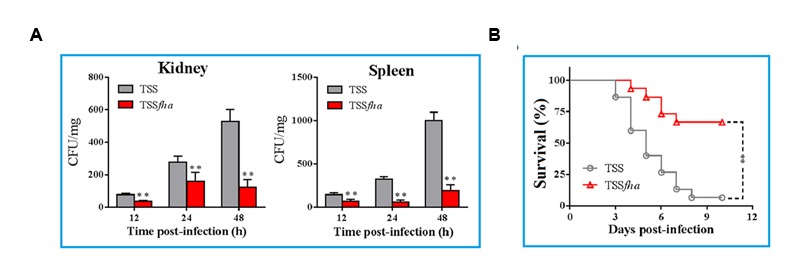
***In vivo* infectivity of *Pseudomonas fluorescens* TSS and TSS*fha*. (A)** TSS and TSS*fha* were inoculated into turbot, and bacterial recovery from kidney (left) and spleen (right) was determined at different times. The results are the means of three independent experiments and presented as mean ± SEM. ^∗∗^*P* < 0.01. **(B)** Turbot were infected with TSS and TSS*fha*, and the fish were monitored daily for mortality and survival for 20 days (only 12 days are shown in the figure). The results are the means of three independent experiments. Significance between the survivals of wild type- and mutant-infected fish was determined with logrank test. ^∗∗^*P* < 0.01.

**FIGURE 9:**
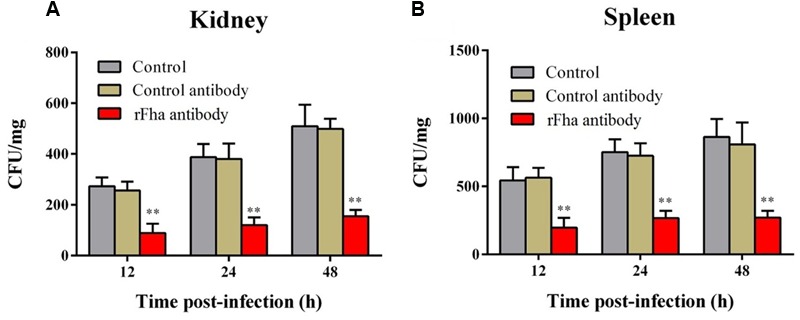
**Effect of rFha antibody on *Pseudomonas fluorescens* infection.** Turbot were infected with TSS in the presence or absence of rFha antibody, control antibody, or PBS (control). Bacterial recovery from kidney **(A)** and spleen **(B)** was determined at different hours post-infection. The results are the means of three independent experiments and presented as mean ± SEM. ^∗∗^*P* < 0.01.

*In vivo* infection assay showed that following inoculation into turbot, TSS disseminated into and multiplied in kidney and spleen, in which the numbers of TSS increased rapidly with time (**Figure 8A**). In contrast, in turbot inoculated with TSS*fha*, the bacterial numbers recovered from kidney and spleen were significantly lower than that from TSS-infected fish at 12, 24, and 48 h post-infection. Similar results were obtained in repeated infection analyses with TSS, TSS*fha*, and TSSΔ*fha* in parallel, which showed that at 12, 24, and 48 h, the amounts of TSSΔ*fha* in kidney and spleen were comparable to those of TSS*fha* and were significantly lower than those of TSS (Supplementary Figure S8). Consistent with these observations, the survival rate of TSS*fha*-infected fish (66.7%) was significantly higher than that of TSS-infected fish (6.7%; **Figure 8B**). To further investigate the importance of Pf_Fha_ to host infection, turbot were infected with TSS in the presence of anti-rFha antibody, and bacterial dissemination in and colonization of kidney and spleen were subsequently determined. The results showed that at 12, 24, and 48 h post-infection, bacterial recoveries from the fish infected with TSS plus rFha antibody were significantly lower than those from the fish infected with TSS alone or with TSS plus control antibody (**Figure 9**).

### Immunoprotection Induced by rFha

**FIGURE 10:**
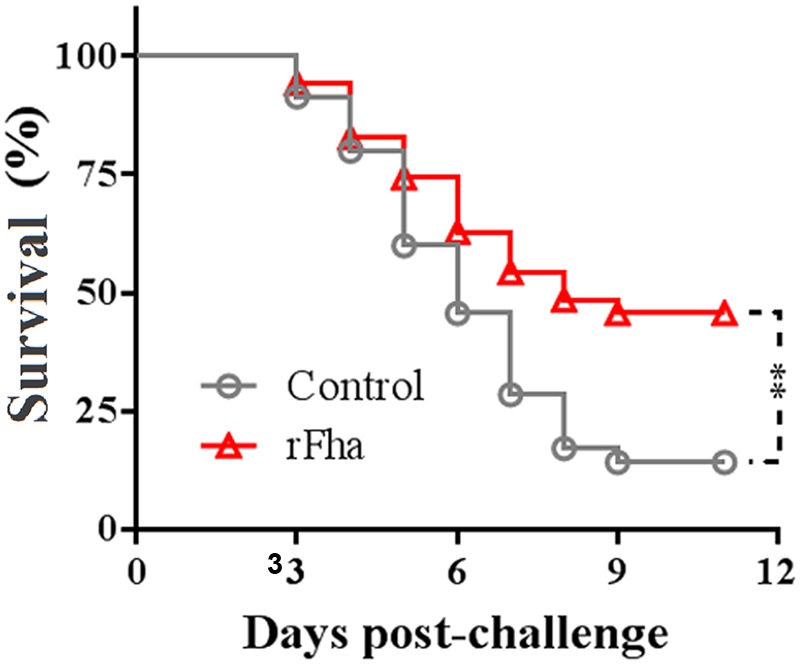
**Protective effect of rFha as a vaccine.** Turbot were vaccinated with or without (control) rFha and challenged with *P. fluorescens* TSS at 1 month post-vaccination. The fish were monitored daily for survival. Significances between the survivals of the vaccinated fish and the control fish were determined with logrank test. ^∗∗^*P* < 0.01.

Since, as shown above, Pf_Fha_ was a secreted protein essential to host infection, we examined the immunoprotective potential of rFha in a model of turbot. For this purpose, turbot were vaccinated with rFha and challenged with TSS at 1 month post-vaccination. The fish were subsequently monitored for mortality and survival. The results showed that the survival rate of the vaccinated fish was 45.7%, which was significantly higher than that of the control fish (14.3%; **Figure 10**). The RPS of the vaccinated fish was 36.7%.

## Discussion

Our previous study has shown that in TSS, iron limitation altered the expression of a large amount of cytoplasmic and membrane associated proteins involved in diverse functions (Sun and Sun, 2015). In the present study, we found that Pf_Fha_ was a secreted protein whose expression was upregulated during iron starvation. The presence of Pf_Fha_ in the extracellular milieu is in agreement with the previous reports that FHA is both surface associated and secreted (Weiss and Hewlett, 1986; Locht et al., 1993). Consistently, Pf_Fha_ contains an N-terminal putative signal peptide and is predicted to be an extracellular protein. Pf_Fha_ also contains a hemagglutination activity domain, which is usually found near the N terminus of FHA and heme/hemopexin-binding proteins (Ruer et al., 2008).

Reports have indicated that in *Xanthomonas campestris* pv. *vesicatoria*, mutation of *fha* significantly reduced swarming motility (Choi et al., 2013), whereas in *X. axonopodis* pv. *citri*, mutation of *fha* resulted in faster swarming (Gottig et al., 2009); but neither of these phenotypes related to flagella. In our study, TSS*fha* displayed very little motility compared to the wild type and, surprisingly and interestingly, exhibited no apparent flagellum. To our knowledge, this is the first evidence that suggests a link between FHA and flagella. These observations imply that Pf_Fha_ is possibly involved in the normal synthesis/transport of flagella. The lack of flagella may also to some degrees account for the reduced self-aggregation observed with TSS*fha*, since it has been reported that in *E. coli* and *Xylella fastidiosa*, autoaggregation is influenced by surface structures such as type I fimbriae and type IV bundle-forming pili (Ulett et al., 2006; De La Fuente et al., 2008). For many pathogens, motility is often intimately linked to pathogenicity by complex regulatory networks (Josenhans and Suerbaum, 2002). In *Burkholderia pseudomallei*, flagella are necessary virulence determinants during intranasal and intraperitoneal infection of mice (Chua et al., 2003). In the coral pathogen *Vibrio coralliilyticus*, flagellar mutation disables coral attachment, chemotaxis, and host infection (Meron et al., 2009). Considering these observations, it is likely that the motility/flagella-defectiveness of TSS*fha* may contribute at least in part to the attenuated virulence of this mutant.

Many studies have indicated a close relationship between biofilm and bacterial pathogenicity. In Pseudomonas aeruginosa, biofilm production significantly facilitates bacterial infection (Bjarnsholt et al., 2010); in Xanthomonas campestris, biofilm is required for full virulence to plants (Dow et al., 2003); in methicillin-resistant Staphylococcus aureus, the biofilm-forming capacity is crucial for intracellular persistence and chronic infections (Oyama et al., 2016). In X. axonopodis pv. citri, B. pertussis, and A. baumannii, it is known that fha mutation reduced biofilm formation (Gottig et al., 2009; Serra et al., 2011; Astaneh et al., 2014). Likewise, we observed a significant reduction in biofilm production by TSSfha. Accumulating evidences indicate that the motility of bacteria plays an important role in biofilm development (Wood et al., 2006; Meshcheryakov et al., 2013). For example, E. coli lacking flagella or possessing paralyzed flagella is defective in biofilm growth (Wood et al., 2006; Houry et al., 2010), and Edwardsiella tarda with impaired flagella exhibited reduced biofilm production (Xu et al., 2014). Biofilms are multicellular aggregates of bacteria bound by a matrix of extracellular polymers (O’Toole et al., 2000; Branda et al., 2006; Monds and O’Toole, 2009). The matrix of biofilm not only allows the bacteria to cohere to one another but also adhere to solid surfaces (Flemming and Wingender, 2010). In our study, we found that while the wild type TSS was able to form a matrix of fiber-like structures on the solid surface of a cover slip, TSSfha completely lost this capacity. It is possible that the matrix formed by the fiber structures connects the bacterial cells into a network and assists cellular adhesion to the attached surface, thus promoting biofilm growth.

Filamentous hemagglutinin plays a crucial role in host interaction by involvement in the adherence to host surface and hemagglutination (Rojas et al., 2002; Guilhabert and Kirkpatrick, 2005; Gottig et al., 2009; Serra et al., 2011). A previous study showed that in *Erwinia chrysanthemi*, mutation of *hecA*, the hemagglutinin homolog of *fha*, decreased the ability to attach to leaves and form aggregates on them (Rojas et al., 2002). In our study, TSS*fha* was markedly impaired in attachment to FG cells, suggesting that Pf_Fha_ was vital to the binding of the bacteria to host cells. Consistent with its structural prediction, TSS exhibited apparent hemagglutination activity, which, however, was absent in TSS*fha*, suggesting that as observed previously in bacteria such as *B. pertussis* (Colombi et al., 2004), Pf_Fha_ functions as a hemagglutinin.

Filamentous hemagglutinin as a key virulence factor has been reported in both plant and animal pathogens including *E. chrysanthemi, B. pertussis*, and *A. baumannii* (Rojas et al., 2002; Astaneh et al., 2014; Romero et al., 2014). In our study, we found that compared to the wild type, TSS*fha* exhibited a distinct reduction in serum survival, which is the first observation of an association between bacterial FHA and serum resistance. Given the fact that Pf_Fha_ is a surface protein of the bacteria, which enables it to be in direct contact with host factors, it is possible that, as observed previously with other bacterial factors (Hallström et al., 2013; Bernhard et al., 2014), Pf_Fha_ may be able to interact with, and consequently sequester or inactivate, serum components essential to the functioning of the complement system. *In vivo* study showed that the tissue dissemination and mortality-inducing capacities of TSS*fha* were markedly weakened. Consistently, the presence of rFha antibody, which most likely interacted with and thus blocked the function of the natural Pf_Fha_, significantly reduced the dissemination and colonization of TSS in turbot tissues. Together these results indicate that Pf_Fha_ is required for the full virulence of TSS during the process of host infection.

In *B. pertussis*, FHA is considered one of the most important immunogens, because it is the major secreted protein and mediates host-pathogen interaction (van den Berg et al., 1999; Balder et al., 2007). Similarly, purified FHA protein confers protection against *Bordetella bronchiseptica* in mice (Goebel et al., 2009). In our study, we found that when used as a subunit vaccine, rFha was able to induce a significant protection on immunized turbot, suggesting a potential of rFha as a vaccine candidate for the control of *P. fluorescens* in agriculture. These results are also in line with the secreted nature of Pf_Fha_ and with the involvement of Pf_Fha_ in host interaction.

In summary, we in this study provide a systematic analysis of the biological properties of *P. fluorescens* FHA associated with pathogenicity. We found for the first time that mutation of bacterial FHA affects flagella formation, extracellular matrix production, and serum survival. Like the FHA of other bacteria, *P. fluorescens* Pf_Fha_ is required for adhesion to host cells, hemagglutination, biofilm formation, mortality, and pathogenicity. In addition, *P. fluorescens* Pf_Fha_ in the form of recombinant protein can induce protective immunity in fish. These results add new insights into the function of bacterial FHA.

## Author Contributions

LS and Y-YS conceived and designed the experiments; Y-YS performed the experiments; Y-YS and HC analyzed the data; Y-YS and LS wrote the manuscript. All authors read and approved the final manuscript.

## Conflict of Interest Statement

The authors declare that the research was conducted in the absence of any commercial or financial relationships that could be construed as a potential conflict of interest.
