# Supplementary material for: Pseudomonas fluorescens Filamentous Hemagglutinin, an Iron-Regulated Protein, Is an Important Virulence Factor that Modulates Bacterial Pathogenicity
Source: Front Microbiol. 2016 Aug 23;7:1320. doi: 10.3389/fmicb.2016.01320 (PMC4993755; doi:10.3389/fmicb.2016.01320)
Supplement: Supplementary file 1 [file Table_1.PDF]

Supplementary Material

Pseudomonas fluorescens filamentous hemagglutinin, an iron-regulated protein, is an important virulence factor that modulates bacterial pathogenicity

Yuan-yuan Sun, Heng Chi, Li Sun*

*** Correspondence:** Li Sun: lsun@qdio.ac.cn

Supplemental Tables

Table S1. Summary of the differentially expressed proteins.

Spot no.[a]	NCBI no.	Protein name	Abrr.	MASCOT score[b]	Theoretical pI/Mr (kD)	Coverage (%)[c]	Fold ratio TSS + DP/TSS[d] (mean ± SD)[e]
1	gi|387894385	Hypothetical protein PflA506_3215 [*Pseudomonas fluorescens* A506]		424	5.23/27.51	38	∞
2	gi|387896505	ATP synthase F1 subunit beta [*Pseudomonas fluorescens* A506]	atpD	972	4.92/49.44	77	0.28±0.07
3	gi|387893805	serine protease B [*Pseudomonas fluorescens* A506]	pspB	420	5.38/107.07	21	0.09±0.01
4	gi|387894927	outer membrane protein transport protein,OMPP1/FadL/TodX family [*Pseudomonas fluorescens* A506]		1,140	6/45.60	70	0.41±0.11
5	gi|387894899	flagellin domain-containing protein [*Pseudomonas fluorescens* A506]		302	5.06/57.24	35	0.27±0.05
6	gi|387895327	adhesin/hemagglutinin, HecA family [*Pseudomonas fluorescens* A506]		438	6.23/ 171.08	13	2.97±0.43
7	gi|387892089	superoxide dismutase Mn [*Pseudomonas fluorescens* A506]	SodA1	111	5.59/22.33	12	26.6±6.03
8	gi|388006393	hypothetical protein PseBG33_1367 [*Pseudomonas synxantha* BG33R]		398	5.26/34.77	38	2.53±0.45
9	gi|387894537	lipoprotein [*Pseudomonas fluorescens* A506]		384	5.75/51.72	19	0.38±0.08
10	gi|489286780	heme acquisition protein HasAp [*Pseudomonas fluorescens*]		134	4.26/21.06	10	∞
11	gi|387893049	dihydrolipoyl dehydrogenase [*Pseudomonas fluorescens* A506]		221	5.93/50.13	11	∞
12	gi|387895731	serine hydroxymethyltransferase [*Pseudomonas fluorescens* A506]	glyA_1	80	5.75/45.26	14	∞
13	NA[f]	NA	NA	NA	NA	NA	0.36±0.12
14	NA	NA	NA	NA	NA	NA	0.22±0.04
15	NA	NA	NA	NA	NA	NA	0.28±0.06

[a] Spot ID represents the number on the 2-DE gels.

[b] MOWSE score is -10 log (p), where p is the probability that the observed match is a random event. Based on the NCBInr database using the MASCOT searching program as MS/M S data. Scores greater than 65 are significant ($p < 0.05$).

[c] Number of amino acids spanned by the assigned peptides divided by the protein sequence length.

[d] TSS + DP/TSS, in the presence and the absence of 2,20-dipyridyl.

[e] Mean, the average protein abundance ratio for three paired samples. SD means the standard deviation of protein abundance ratios of one certain spot of three paired samples.

[f] Not analyzed.
