# Supplementary material for: Pseudomonas fluorescens Filamentous Hemagglutinin, an Iron-Regulated Protein, Is an Important Virulence Factor that Modulates Bacterial Pathogenicity
Source: Front Microbiol. 2016 Aug 23;7:1320. doi: 10.3389/fmicb.2016.01320 (PMC4993755; doi:10.3389/fmicb.2016.01320)

Supplementary Material

Pseudomonas fluorescens filamentous hemagglutinin, an iron-regulated protein, is an important virulence factor that modulates bacterial pathogenicity

Yuan-yuan Sun, Heng Chi, Li Sun*

*** Correspondence:** Li Sun: lsun@qdio.ac.cn

Supplemental Figures

Figure S1. Schematic representations of the structural domains of Pf_{Fha}. The domains are shown in different colors: green, signal peptide; red, haemagglutination activity domain.

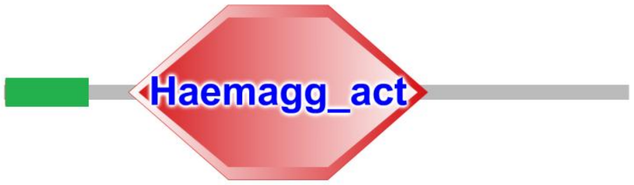

Figure S2. Motility and flagella formation of *Pseudomonas fluorescens* TSS and TSSΔ*fha*. (A) TSS (left) and TSSΔ*fha* (right) were cultured in LB medium to an OD_{600} of 1.0, and 5 μl cell suspensions were spotted onto the centre of swimming plates containing LB medium plus 0.3% (w/v) agar. The plates were incubated at 28 ℃ for 2 days. (B) TSS (left) and TSSΔ*fha* (right) were cultured in LB agar plates and examined with a transmission electron microscope. Scale bar, 1μm. The results represent one of three independent experiments.

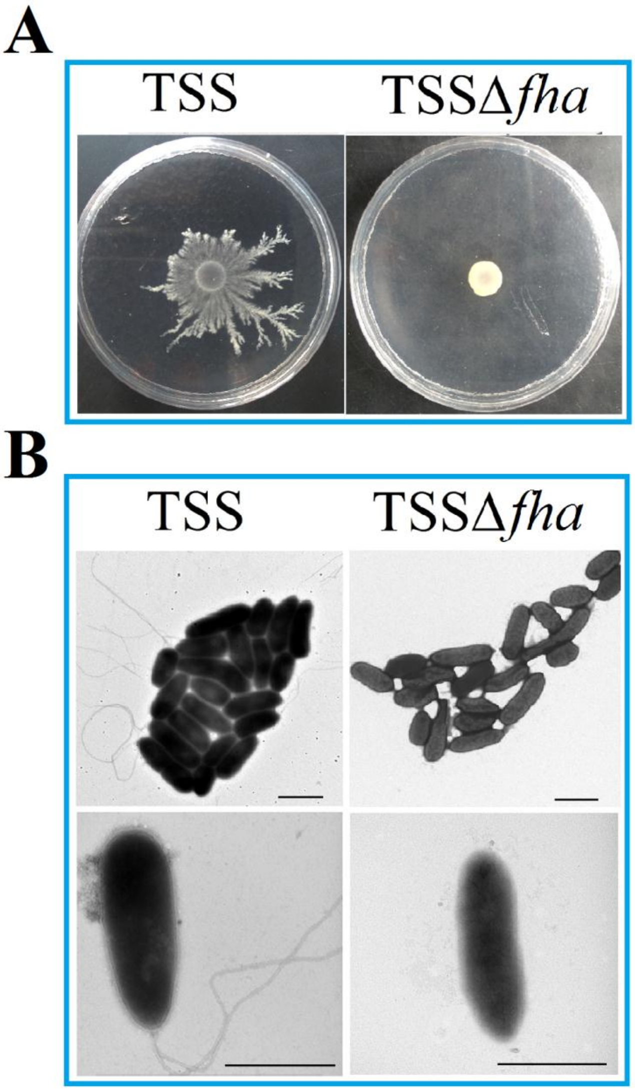

Figure S3. Growth profiles of *Pseudomonas fluorescens* TSS, TSS*fha*, and TSSΔ*fha*. The bacteria were cultured in LB medium supplemented with (B) or without (A) 2,2’-dipyridyl (DP), and cell density was determined at various time points. Data are the means of three independent assays and presented as means ± SEM.

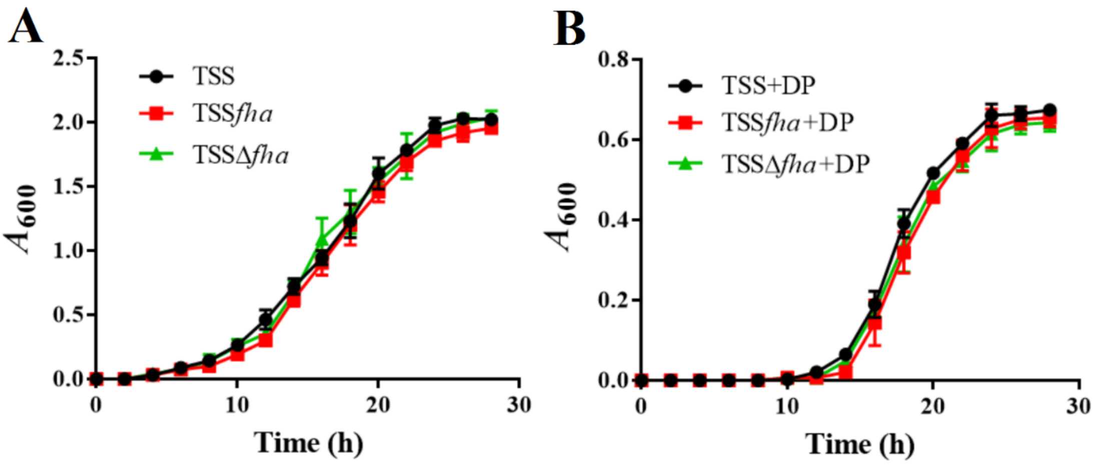

Figure S4. Biofilm formation of *Pseudomonas fluorescens* TSS, TSS*fha*, and TSSΔ*fha*. The bacteria were grown in LB medium overnight at 28 ℃ in a 96-well culture plate, and the biofilm formed was stained with crystal violet. Stained biofilms were dissolved in 30% acetic acid, and the optical density at OD_{570} was recorded. Data are the means of three independent assays and presented as means ± SEM. **P < 0.01.

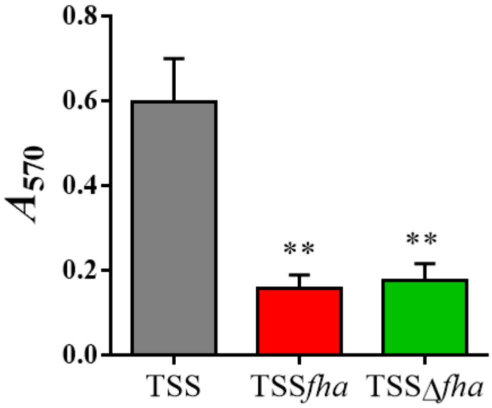

Figure S5. Expression of Pf_{Fha} on bacterial surface. *Pseudomonas fluorescens* TSS was incubated with anti-rFha antibody (A and B) or control antibody (D and E). The bound antibody was detected with FITC-labeled secondary antibody. The cells were stained with DAPI and observed under a microscope with fluorescence. C, a merge of A and B; F, a merge of D and E. Magnification: 40×10, scale bars: 10 μm.

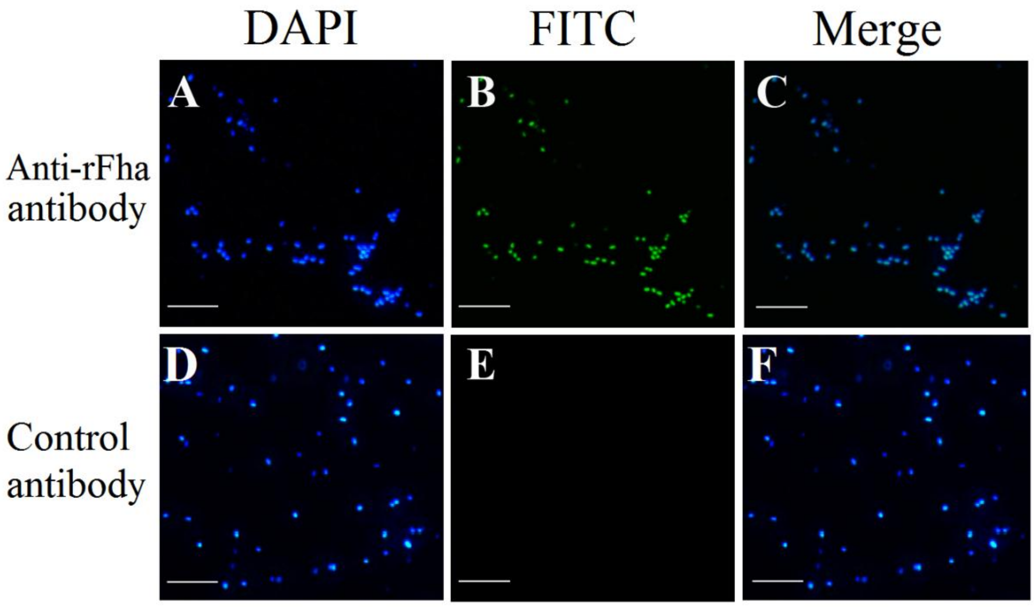

Figure S6. Adhesion of *Pseudomonas fluorescens* TSS and TSS*fha* to host cells. TSS and TSS*fha* were incubated with FG cells for various hours, and the number of cell-bound bacteria was determined by plate count. Data are the means of three independent experiments and are presented as means ± SEM. ***P* < 0.01.

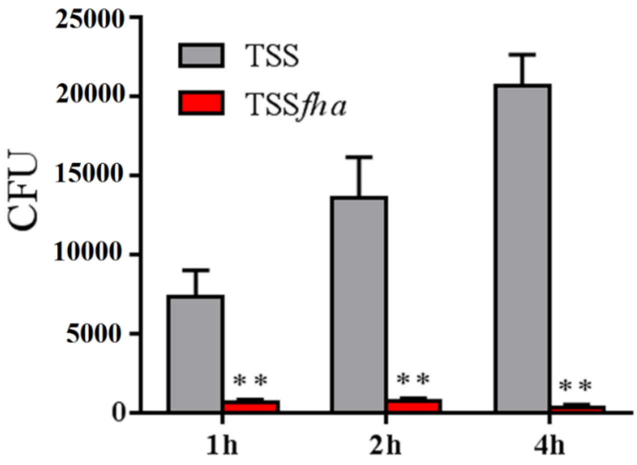

Figure S7. SDS-PAGE analysis of purified recombinant proteins. Purified rFha (lane 2) was analyzed by SDS-PAGE and viewed after staining with Coomassie brilliant blue R-250. Lane 1, protein markers.

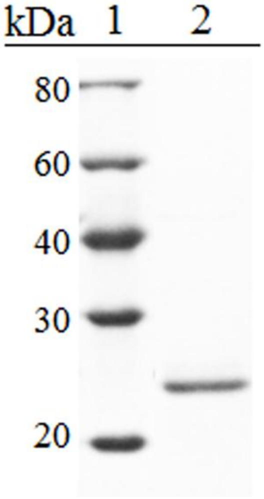

Figure S8. *In vivo* infectivity of *Pseudomonas fluorescens* TSS, TSS*fha*, and TSSΔ*fha*. TSS, TSS*fha*, and TSSΔ*fha* were inoculated into turbot, and bacterial recovery from kidney (A) and spleen (B) was determined at different times. The results are the means of three independent experiments and presented as means ± SEM. *$P < 0.05$, **$P < 0.01$

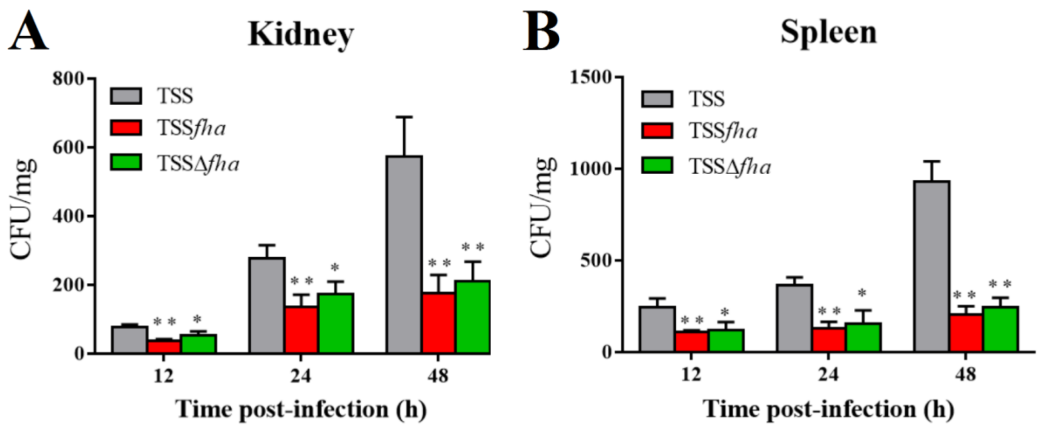

Supplement: Supplementary file 2 [file Image_1.PDF]
